# Study of stability of time-domain features for electromyographic pattern recognition

**DOI:** 10.1186/1743-0003-7-21

**Published:** 2010-05-21

**Authors:** Dennis Tkach, He Huang, Todd A Kuiken

**Affiliations:** 1 Neural Engineering Center for Artificial Limbs, Rehabilitation Institute of Chicago, 345 E. Superior Street, Suite 1309, Chicago, IL, 60611, USA; 2 Committee on Computational Neuroscience, University of Chicago, 1027 E 57th Street, Room 202, Chicago IL, 60637, USA; 3 Department of Electrical, Computer, and Biomedical Engineering, University of Rhode Island, 4 E. Alumni Ave Kelly A-116, Kingston, RI, 02881, USA; 4 Department of Physical Medicine and Rehabilitation, Northwestern University, Chicago, IL, 60611, USA

## Abstract

**Background:**

Significant progress has been made towards the clinical application of human-machine interfaces (HMIs) based on electromyographic (EMG) pattern recognition for various rehabilitation purposes. Making this technology practical and available to patients with motor deficits requires overcoming real-world challenges, such as physical and physiological changes, that result in variations in EMG signals and systems that are unreliable for long-term use. In this study, we aimed to address these challenges by (1) investigating the stability of time-domain EMG features during changes in the EMG signals and (2) identifying the feature sets that would provide the most robust EMG pattern recognition.

**Methods:**

Variations in EMG signals were introduced during physical experiments. We identified three disturbances that commonly affect EMG signals: EMG electrode location shift, variation in muscle contraction effort, and muscle fatigue. The impact of these disturbances on individual features and combined feature sets was quantified by changes in classification performance. The robustness of feature sets was evaluated by a stability index developed in this study.

**Results:**

Muscle fatigue had the smallest effect on the studied EMG features, while electrode location shift and varying effort level significantly reduced the classification accuracy for most of the features. Under these disturbances, the most stable EMG feature set with combination of four features produced at least 16.0% higher classification accuracy than the least stable set. EMG autoregression coefficients and cepstrum coefficients showed the most robust classification performance of all studied time-domain features.

**Conclusions:**

Selecting appropriate EMG feature combinations can overcome the impact of the studied disturbances on EMG pattern classification to a certain extent; however, this simple solution is still inadequate. Stabilizing electrode contact locations and developing effective classifier training strategies are suggested to further improve the robustness of HMIs based on EMG pattern recognition.

## Introduction

Electromyographic (EMG) signals represent neuromuscular activity and are effective biological signals for expressing movement intent for external device control. EMG-based human-machine interfaces (HMIs) have been widely applied in biomedicine, industry, and aerospace. In the field of rehabilitation engineering, EMG signals are one of the major neural control sources for powered upper-limb prostheses [1,2], powered orthoses/exoskeletons [3,4], rehabilitation robots [5,6], robotic wheelchairs [7], and assistive computers [8].

Various EMG signal processing algorithms have been used to decipher movement intent. Simple HMI systems employ methods such as computing root mean square (RMS) to estimate the EMG magnitude. When the EMG magnitude is above a set value, the user's movement intent is identified, which triggers the HMI system to drive an external device. Such algorithms have been used in robotic devices [5-7] and upper-limb prostheses [9], but with limited function. For example, EMG signals from a residual pair of agonist/antagonist muscles were used to proportionally drive a prosthetic joint [9]. Each EMG signal controlled motor rotation in one direction. Although such prostheses have been widely used in clinics, they do not provide sufficient information to reliably control more than one degree of freedom. In addition, users must be trained to avoid co-contracting the two muscles in order to drive the artificial joints smoothly.

EMG pattern recognition is an advanced, intelligent signal processing technology and has been proposed as a potential method for reliable user intent classification [8,10]. Beyond signal magnitude, a typical pattern recognition algorithm extracts a set of features that characterize the acquired EMG signals and then classifies the user's intended movement for external device control. The benefit of pattern recognition algorithms are that they can increase the neural information extracted from EMG signals using a small number of monitored muscles and allow intuitive control of external devices. Previous studies have evaluated the ability of various EMG features and classifiers to recognize user intent [6,8,11-14]. These studies were mainly done on able-bodied subjects or on subjects with transradial amputations. The results demonstrated over 90% classification accuracy for either offline or online testing. The comparison of classification accuracies resulting from utilization of different types of classifiers and EMG features demonstrated that the type of classifier used does not significantly affect the classification performance, while the choice of features has a significant impact on classification performance [11-13].

Although these previous studies reported high classification accuracies in single-session experiments conducted in research laboratories, the robustness over time of HMIs based on EMG pattern recognition has rarely been evaluated [15]. Our research group attempted to implement HMIs based on EMG pattern recognition in clinics. In our experience, the performance of these systems can degrade within hours after initial classifier training [16]. This significantly challenges the clinical application of such systems. This performance degradation could be the result of EMG signal variations caused by undesired disturbances. One simple solution is to identify EMG features that are not only insensitive to the changes in EMG signals caused by these disturbances, but also maintain a high level of class separability. Zardoshti-Kermani et al. [12] defined high-quality features as those that produce maximum class separability, robustness, and less computational complexity. In their study, robustness of features was tested by a repeat measurement of the classifier's performance with artificially added white noise. However, the factors affecting EMG pattern recognition the most may be more complex than additional noise and might be due to physical and physiological changes that directly interfere with the EMG signal sources.

In this study, we investigated the general impact of EMG signal variations on 11 commonly used EMG features and identified the most robust EMG feature sets for reliable EMG pattern recognition. To keep the computational complexity low, our investigation focused only on time-domain (TD) features that do not require additional signal transformation. Additionally, instead of using computer simulation, we collected EMG data from human subjects with three changing physical or physiological conditions: EMG electrode location change (physical change of electrodes), muscle contraction effort (cognitive variations in users), and muscle fatigue (electrophysiological changes in users). These three factors are common disturbances of EMG signal sources in EMG pattern recognition.

*Changing electrode location*: Unlike the self-adhesive EMG electrodes used in a laboratory, the EMG electrodes used in prostheses or exoskeletons are usually metal contacts mounted on the inside wall of a socket or robotic limb. Sliding motion between the rigid structure and the user's limb causes shifts in the electrode contact location and therefore affects the recorded EMG signals [17].

*Variability of muscle contraction effort*: Pattern recognition is composed of two procedures: training and testing. During the training procedure, the classifier must "learn" the patterns of EMG signals generated when the user performs different tasks. The EMG classifier can then be used to identify user intent. However, maintaining the same effort of muscle contraction while controlling an external device as that used when training the classifier could be difficult. It is well known that the muscle contraction force determines the number and type of recruited muscle fibers, thus directly affecting the magnitude and frequency of surface EMG signals [18].

*Muscle fatigue*: Muscle fatigue is another factor that influences the EMG signal [19,20]. Muscle fatigue is common for users with neuromotor deficits, even with the assistance of robots or exoskeletons. Amputee users also experienced fatigue after several hours of myoelectric prosthesis use, mostly due to the sustained muscle contraction.

The outcomes of this study could inform the design of more robust and clinically viable EMG pattern recognition systems for specific rehabilitation applications and eventually benefit individuals with motor deficits.

## Methods

### Participants and Experimental Protocol

This study was approved by the Institutional Review Board at Northwestern University. Eight able-bodied subjects (four male and four female, 35 ± 15 years in age) participated in the study and provided written and informed consent.

**Figure 1:**
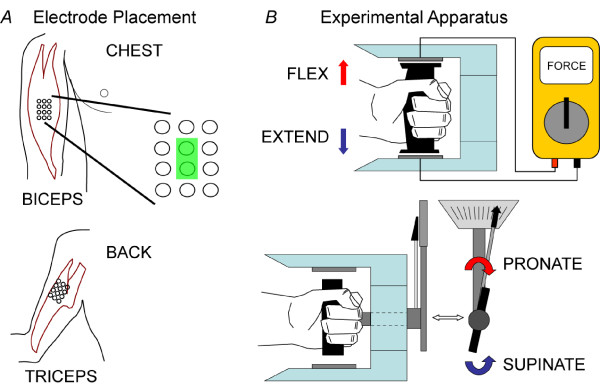
**Experimental apparatus and the placement of electrodes**. (A) Electrode grids were placed on the biceps and triceps muscles of the participants. Single differential EMG signals were obtained by subtracting data from two longitudinally neighboring electrodes (e.g. green box). (B) Subjects grip a handle that is pressed upward or downward against the enclosure of the apparatus to achieve flexion or extension, respectively. Force sensors encased in the upper and lower enclosure provide force feedback. To achieve pronation or supination, subjects twist a handle. The handle is attached to a torque wrench providing the subjects with torque feedback.

Two four-by-three grids of monopolar surface electrodes were placed on each subject, one over the biceps muscle and one over the triceps muscle (Figure 1A). Each monopole Ag/AgCl electrode (TMS International B.V., the Netherlands) was circular with a diameter of 10 mm. The center-to-center distance between two poles was 15 mm. Before electrode placement, the skin was shaved, lightly abraded, and cleaned with alcohol. Conductive gel was applied to each monopole. The center of the electrode grids were positioned over the anatomical locations described by Delagi and Perotto [21]. A reference electrode was placed on the abdomen of each subject. The subjects were asked to perform four types of isometric contractions with their preferred arm: elbow flexion, elbow extension, forearm pronation, and forearm supination. They were also asked to complete resting trials. An experimental apparatus (Figure 1B) was constructed to maintain a consistent arm posture and normalize the level of effort exerted by all subjects. Subjects sat comfortably in front of a desk with their elbow resting on an armrest, such that their elbow joint was at a right angle and their hand was level with the top of the desk. Their hand gripped the handle of the experimental apparatus. Elbow flexion and extension were performed by pressing the handle upward or downward against force sensors within the upper or lower enclosure of the apparatus, respectively. Pronation and supination were achieved by gripping a handle connected to a torque wrench and rotating the forearm against the resistance of the device while maintaining proper arm posture. No effort was required for the subjects to maintain their nominal posture in the experimental apparatus.

To study the effect of different levels of muscle contraction effort on classifier performance, we defined two different effort levels--high and low. At the beginning of each experiment, subjects were asked to perform each of the four actions at their own pace and maintain maximal voluntary contractile force (MVC) for five seconds. Low and high effort levels were defined as 25% and 65% of MVC, respectively, in congruence with effort protocols seen in literature [20,22]. Although 25% to 65% effort levels are high compared to the effort required by able-bodied subjects to naturally move a joint without load, powered prostheses, wheelchairs, or exoskeletons are usually driven by EMG signal amplitudes (or muscle contraction effort), and therefore patients with motor deficits use these effort levels to drive these machines. Once MVC was established, subjects were asked to perform flexion, extension, supination, or pronation at their own pace and to hold the contraction at the defined effort level for 5 s. Subjects were given feedback on their effort level via either the force sensors or the torque wrench (Figure 1B).

In order to study the effect of muscle fatigue on EMG features, we instructed the subjects to perform isometric contractions that induced short-term muscle fatigue. Subjects were asked to maintain an isometric contraction of the respective muscle at low effort (25% MVC) for 90 seconds [23,24]. All subjects verbally reported muscle soreness and presented some difficulties in maintaining the required amount of constant force at the end of this session. The EMG signals measured after this 90 seconds contraction were from fatigued muscles.

The experiment was divided into ten trials--a baseline/rest trial and nine action trials. During the first trial, the subjects remained relaxed for 2 min. while baseline EMG activity was recorded. Each of the remaining trials consisted of 10 isometric contractions, either 5 flexions and 5 extensions, or 5 pronations and 5 supinations. The type of action and desired effort level were specified randomly within each trial. For each action, subjects were instructed to maintain a target level of contraction--either low or high effort, depending on the trial--for 5 s, with 5 s breaks between low effort contractions and 1.5 min. breaks between high effort contractions to avoid muscle fatigue [19,23]. During the first seven of the nine action trials the subjects were instructed to perform isometric contractions at either low or high levels of effort while the muscles remained unfatigued. The last two of the nine action trials required the subjects to perform only low effort actions while the muscles were in a fatigued state. A rest was allowed between trials.

### EMG Data Collection and Pre-Processing

The Refa System (TMS International B.V., the Netherlands) was used to acquire the EMG signals. The monopolar analog signals were low-pass filtered with a 625 Hz cut-off frequency and pre-amplified with the gain of 60 dB. The common mode was removed by subtracting the average of the connected monopole signals. The EMG signals were digitally sampled at 2500 Hz and band-pass filtered from 15 to 450 Hz using a digital, eighth-order Butterworth filter. The data coinciding with muscle contractions were manually segmented and concatenated based on the type of intended movement [25]. Manual data segmentation allowed us to select transient EMG signals in the initial movement state, compared to automatic method. Note that the data segmentation was not required in real-time EMG pattern recognition. Single differential EMG signal (bipolar) recordings from longitudinally neighboring electrodes were subtracted from each other (see, e.g., Figure 1A). Single differential EMG signals are referred to below as EMG signal channels.

### Investigation of Time-Domain Features

Eleven frequently suggested time-domain features with high computational efficiency [10,12,15,26-28] for real-time EMG pattern recognition were assessed. These features were extracted within an N-sample analysis time window.

#### Mean Absolute Value (mAV)

This feature is the mean absolute value of signal *x *in an analysis time window with N samples. *x*_*k *_is the k^th ^sample in this analysis window.(1)

#### Zero Crossings (ZC)

ZC is the number of times signal *x *crosses zero within an analysis window; it is a simple measure associated with the frequency of the signal. To avoid signal crossing counts due to low-level noise, a threshold *ε *was included (*ε *= 0.015 V) [27]. The ZC count increased by one if(2)

#### Slope Sign Changes (slopeSign)

Slope sign change is related to signal frequency and is defined as the number of times that the slope of the EMG waveform changes sign within an analysis window. A count threshold *ε *was used to reduce noise-induced counts (*ε *= 0.015 V) [27]. The *slopeSign *count increased by one if(3)

#### Waveform Length (waveLen)

This feature provides a measure of the complexity of the signal. It is defined as the cumulative length of the EMG signal within the analysis window:(4)

#### Willison Amplitude (wAmp)

This feature is defined as the amount of times that the change in EMG signal amplitude exceeds a threshold; it is an indicator of the firing of motor unit action potentials and is thus a surrogate metric for the level of muscle contraction [12]. A threshold between 50 and 100 mV has been reported in the literature [12]. In this study, the threshold *ε *was defined for each subject as the EMG signal value that had a 50% probability of occurrence as defined by a computed cumulative distribution function for each type of intended movement:(5)

where *f*(*x*) = {1 if *x *> *ε*; 0 otherwise}.

#### Variance (var)

This feature is the measure of the EMG signal's power.(6)

#### v-Order (vOrder)

This metric yields an estimation of the exerted muscle force [12]. The optimal EMG signal processor consists of a pre-whitening filter, a nonlinear detector, a smoothing filter, and a re-linearizer [12]. The nonlinear detector here is characterized by the absolute value of EMG signal to the v^th ^power. The applied smoothing filter is the moving average window. Therefore, this feature is defined as , where E is the expectation operator applied on the samples in one analysis window. One study [12] indicates that the best value for *v *is 2, which leads to the definition of the EMG *v-Order *feature as the same as the square root of the *var *feature.

#### log-Detector (logDetect)

Like the *vOrder *feature, this feature also provides an estimate of the exerted muscle force [12]. The nonlinear detector is characterized as log(|*x*_*k*_|) and the *logDetect *feature is defined as(7)

Aside from the single-value features described above, we also studied three features with multiple dimensions. Each of them captured one or more characteristics of the EMG process. To be consistent, the dimensionality of these features was constrained to nine.

#### EMG Histogram (emgHist)

This feature provides information about the frequency with which the EMG signal reaches various amplitudes [12]. For each subject, a minimum and a maximum voltage value of the EMG signal were determined and used as the data range for a histogram with nine data bins. We refer to this feature as *emgHist*. Although the data range for computing *emgHist *was different among subjects, this did not bias the classification result because the classifier was adaptive to the EMG patterns for individual subjects.

#### Autoregression Coefficient (AR)

This feature models individual EMG signals as a linear autoregressive time series and provides information about the muscle's contraction state. It is defined as(8)

where *a*_*i *_represents autoregressive coefficients, *p *is the AR model order, and *e*_*k *_is the residual white noise [26].

#### Cepstrum coefficients (Ceps)

A cepstrum of a signal is the result of taking the Fourier transform of the decibel spectrum as if it were a signal. This measure provides information about the rate of change in different frequency spectrum bands of a signal. Cepstrum coefficients were derived from the autoregressive model [15] and were computed as(9)

where *a*_*i *_is the *i*^th ^AR coefficient as (8), *c*_*i *_is the *i*^th ^Cepstrum coefficient, *i *is the dimensionality of the model. Note that computing this feature does not require a Fourier transform, and this feature is still considered a time-domain feature.

#### Analysis of Disturbance Impact on EMG Features

The impact of the studied disturbances on individual features and combined features was quantified by the change in classification performance. A simple linear discriminant analysis (LDA) classifier was used because it is a computationally efficient real-time operation and has classification performance similar to more complex algorithms [10,29,30]. One EMG channel from the biceps and one channel from the triceps were input to the LDA classifier to identify five intended movements. For each movement class, the concatenated signals were separated into 150 ms analysis windows with 75 ms (50% of duration) of overlap [25]. EMG features were calculated for each analysis window for each EMG channel. Features for two EMG signals were concatenated into a vector and passed to the LDA classifier. EMG features were further separated into a training data set (to train the classifier) and a testing data set (to evaluate the classifier). The classification performance was quantified by the overall classification accuracy (*CA*):(10)

To investigate feature stability with respect to the three studied disturbances, the training and testing data were organized as follows:

#### Location Stability

The electrode shift was assumed to occur in the same manner as a hypothetical orthotic or prosthetic socket that could rotate clockwise/counterclockwise or slide up/down along a user's arm. To study the effect of electrode shift, the classifier was trained using the channel pairs located in the center of the electrode grids on the biceps and triceps. The classifier was then tested on data from each of four pairs of channels with locations that would coincide with socket shift (up/down) and socket rotation (clockwise/counterclockwise). The extent of the shift was constrained to the neighboring electrode pair: 15 mm shift in each of the four directions.

#### Effort Stability

Based on our clinical experience, users may exert one level of muscle contraction effort while training an EMG classifier but use a different level of effort during real-time testing. The effort stability was studied by training the EMG classifier on data gathered from high-effort actions and testing the classifier on data gathered from low-effort actions, and vice versa. In addition, to explore different training strategies, a classifier was also trained and tested on data of mixed high- and low-effort actions. EMG signals used in this analysis were taken from trials without muscle fatigue. The central pair of electrodes with respect to the electrode grid was used for this analysis.

#### Fatigue Stability

In this analysis, the classifier was trained on trials without muscle fatigue and then tested on data corresponding to trials with muscle fatigue. During clinical testing, muscle fatigue emerges following prolonged usage of EMG pattern recognition systems but does not typically emerge during the training phase. The effort level was set to low, and the central pairs of electrodes on the electrode grids were used.

### Identification of Robust Feature Sets

A robust EMG feature set should exhibit minimal impact from undesired disturbances, yet remain sensitive to the user's intended movements. To quantify the robustness of feature sets under the influence of the studied disturbances, we defined a stability index as follows:(11)

The numerator is the average classification accuracy over N samples; the denominator is the scaled standard deviation. *α *is a scaling factor that limits the influence of the standard deviation on the index value. A robust feature set should produce high average classification accuracy under the disturbance as well as low variance across subjects; therefore, the optimal feature set must provide the highest index value. In this study, *α *was set to 0.2. This value was determined by trial and error. It is noteworthy that the optimal feature set was not sensitive to *α *when *α *was within the range from 0.1 to 0.3. The most robust EMG feature sets were determined for each of the three studied disturbances as well as for the combination of the three studied disturbances.

## Results

### Impact of Disturbances on EMG signals

**Figure 2:**
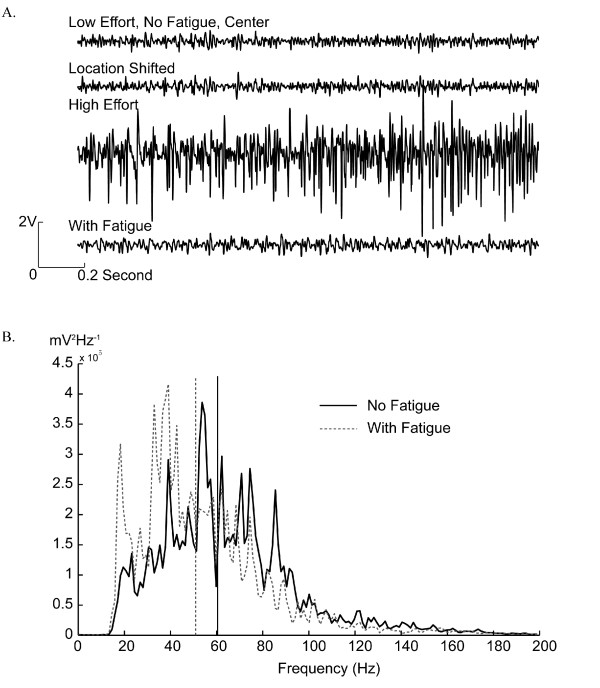
**Examples of recorded EMG signals**. (A) Comparison of raw EMG signals recorded during electrode location shift, effort level change, and muscle fatigue. (B) Comparison of power spectral density (PSD) of EMG signals with and without fatigue. The representative PSD was estimated using sampled data for elbow flexion. The effort level was set to low. Median frequencies are demonstrated by the vertical dashed lines. The median frequency is 60.4 Hz without muscle fatigue and is 50.8 Hz when the muscle is fatigue. The estimated signal power is 1.42 × 10^7 ^mV^2 ^without muscle fatigue and is 1.46 × 10^7 ^mV^2 ^with muscle fatigue.

The impact of electrode location shift, changing effort level, and muscle fatigue on EMG signals recorded from the biceps are shown in Figure 2A. Shifting the electrode location by 15 mm caused a slight change in magnitude in EMG signals. Significantly larger EMG amplitudes were observed with high muscle contraction effort than with low effort. In addition, the EMG signals recorded during muscle fatigue demonstrated an attenuation of the higher frequency components as compared with the EMG signals recorded without muscle fatigue. Figure 2B highlights this observation by comparing the power spectrum density of the EMG signals recorded with and without fatigue; the median frequency was reduced by 9.6 Hz when the muscles were fatigued.

### Impact of Disturbances on Individual Features

**Figure 3:**
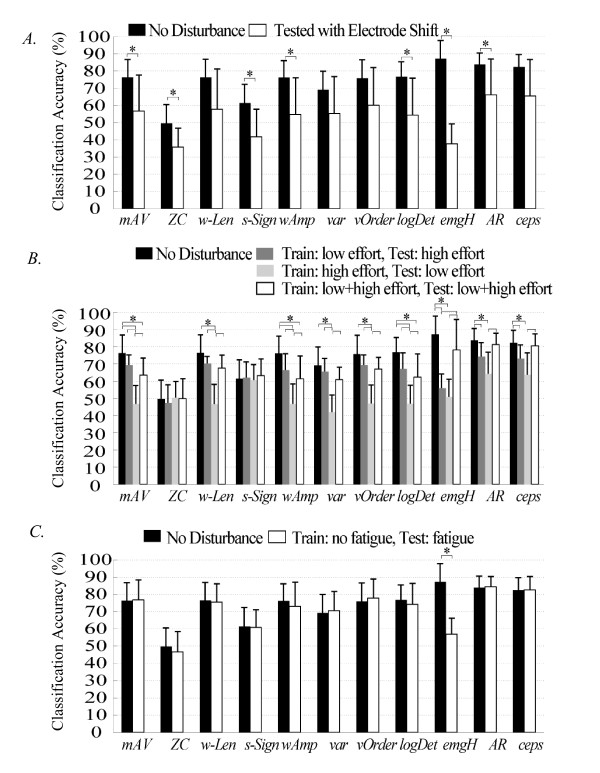
**The effects of (A) location shift, (B) varied muscle contraction effort, and (C) muscle fatigue on the classification performance of individual features**. Each bar indicates the mean value of classification accuracy over 8 subjects. The error bars denote one standard deviation. Stars (*) denote statistically significant differences by one-way ANOVA (*P *<*0.05*).

The effects of the three studied perturbations on individual features are demonstrated in Figure 3. When the electrodes were not shifted, the use of emgHist resulted in the highest mean classification accuracy (87.3%) and the use of *ZC *yielded the lowest mean accuracy (49.7%). Introducing a 15 mm electrode location shift in the testing data led to lower classification accuracy for all of the features (Figure 3A).

Stability of individual features with respect to the level of muscle contraction effort is demonstrated in Figure 3B. Compared with the performance without any disturbances, variation in muscle contraction effort reduced the classification accuracy of all individual features except for *ZC *and *slopSign*. Training the classifier on high-effort data yielded the lowest classification performance for all features except for *ZC*. Training the classifier on low- and mixed-effort data resulted in similar accuracies. Overall, *AR *and *Ceps *were influenced the least and provided relatively high classification accuracies.

Stability of individual features with respect to muscle fatigue is demonstrated in Figure 3C. Muscle fatigue only affected the classification accuracy of the *emgHist *feature.

### The Impact of Disturbances on Feature Combinations

**Figure 4:**
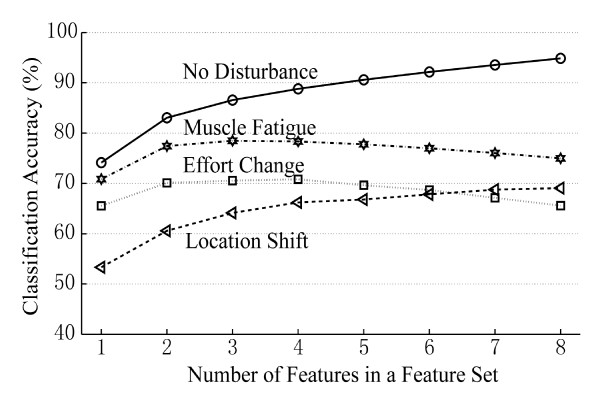
**The change in average classification performance with the number of applied features in a feature set**. Note that the curve of effort stability (dotted line) was derived from the classifier trained on the low-effort data and tested on the high-effort data.

Figure 4 demonstrates the average classification accuracy as a function of the number of combined features during each perturbation. All three disturbances reduced the classification performance of feature combinations. Although muscle fatigue did not significantly affect the classification performance of each individual feature, its impact became visible when combinations of features were used.

Using a feature set with two or more combined features improved EMG pattern classification performance in all studied conditions. In addition, using a combination of four features began to saturate the classification performance when tested with disturbances, which implies that at least four features should be used to reduce the impact of the three disturbances on the EMG pattern recognition performance. Using features sets with five or more combined features increased the computational complexity of pattern recognition and did not result in further improvement of classification accuracy when tested with muscle fatigue and changing effort level. Therefore, the most stable feature set was identified from the combinations of four features.

### Selection of Most Stable Feature Sets

When the number of combined features was limited to four, the feature set with the highest stability index (as in equation 11) with respect to location shift was composed of *var*, *v-Order*, *logDetect*, and *emgHist *features. The use of this optimal feature set produced a 72.6% mean accuracy across the 8 subjects, with a standard deviation of 21.9%. The difference between the accuracy derived from the feature set with the highest stability index and the accuracy derived from the feature set with the lowest index (56.6% ± 22.5%) was not statistically significant (one-way ANOVA, *p *= 0.17). The classification accuracies derived from both feature sets demonstrated a large variation across subjects.

The muscle contraction effort stability was studied using training data from low-effort muscle contractions and testing data from high-effort muscle contractions. The most stable feature set with respect to a changing level of effort consisted of *waveLen*, *slopeSign*, *logDetect *and *AR *features. Using this feature set produced 76.3% ± 8.03% accuracy when averaged across 8 subjects, which was significantly higher than the accuracy (57.9% ± 17.3%) derived from the worst performing feature set (one-way ANOVA, *p *< 0.05).

The most stable feature set with respect to muscle fatigue consisted of *waveLen*, *slopeSign*, *AR *and *Ceps *features, which resulted in 85.6% ± 4.8% accuracy across subjects. The feature set with the lowest index value resulted in 65.1% ± 11.4% accuracy, which was significantly lower than the most stable feature set (one-way ANOVA, *p *< 0.05).

**Figure 5:**
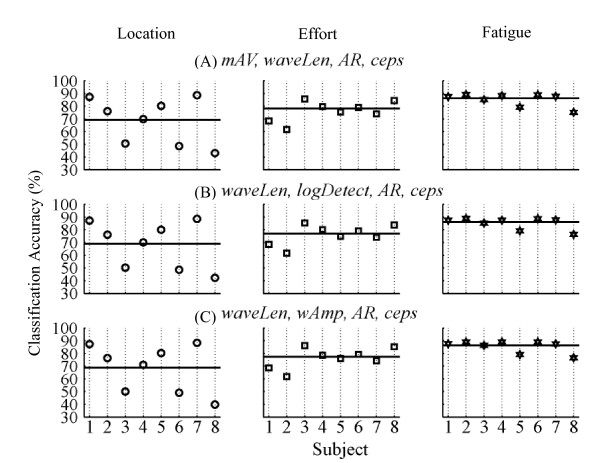
**Performance of the three optimal feature sets under three studied disturbances**. The three feature sets are (A) *mAV, waveLen, AR, and Ceps*; (B)*waveLen, logDetect, AR, and Ceps*; (C)*waveLen, wAmp, AR, and Ceps*. Each graph is divided into three columns. The first column shows classification accuracies for each subject with respect to location stability. The second column shows classification accuracies for each subject with respect to effort stability. Only the classifier trained on low effort data and tested on high effort data was considered. The third column shows classification accuracies with respect to fatigue stability. Horizontal black lines in each column of all the graphs show the mean classifier performance across all subjects for the stability condition.

Lastly, the stability of a feature set with respect to all studied disturbances was of primary interest in our analysis. The stability index of each feature set was calculated across the three studied disturbances and all tested subjects. Note that we only considered the effort level change from low (training) to high (testing). Figure 5 shows the performance of the three EMG feature sets with the highest stability index across the three studied disturbances. All three feature sets produced similar classification performance; the average classification accuracy over 8 subjects was approximatley 70% under electrode location shift, 78% under effort level change, and 87% with muscle fatigue. All three sets shared the features of *waveLen, AR*, and *Ceps*.

## Discussion

Practical usage of EMG pattern recognition demands that performance remains invariant across prolonged periods of time. This requirement translates into the need for an understanding of the consequences of inevitable disturbances, such as a shift in the location of EMG electrodes, variations in muscle contraction effort, and muscle fatigue. It is therefore necessary to identify parameters of the control signal that are robust with respect to these disturbances. Our study achieved two goals in addressing this practical problem: (1) we quantified the performance of EMG features under three physical and physiological disturbances and then (2) attempted to improve the robustness of EMG pattern classification by identifying robust sets of EMG features. The experiments of this study were designed with the aim of examining the stability of EMG features under general variations in EMG signals; the results could inform other HMI design for different specific applications. Note that other HMI system may include different number of EMG electrodes, other type of tested movements, and different classifier, which may have effects on the absolute system accuracy, but little on the relative difference in classification accuracy between the before and the after signal disturbance phase. Since the stability of TD features was measured by the relative change of accuracy after EMG disturbances, the outcome of this study can benefit general EMG-based HMI system design by selecting stable EMG features.

A shift in electrode location greatly diminished the classification accuracies of each individual feature as well as feature combinations. Choosing the most stable combination of four features with respect to electrode location resulted in only 72.6% classification accuracy, which was significantly lower than the average accuracy (~90% in Figure 4) when no disturbance was presented. This result implies that simply selecting proper time-domain EMG feature sets can offer some improvement in classification accuracy, but is inadequate to compensate for the large shifts (15 mm) on the biceps and triceps tested in this study. Physically maintaining electrode location is vital to achieving robustness of EMG pattern classification. Further investigation is required to assess the sensitivity of EMG features to electrode shifts in other muscle areas and shifts smaller than the ones we considered in our study.

Similar results were observed for the level of effort of muscle contractions. Using the feature set with the highest stability index offers improvement in classification accuracy, but cannot effectively negate the impact from the effort level change. Interestingly, the magnitude of the impact due to variability in effort level was considerably influenced by the training strategy used for our classifier. Training the classifier on data from low-effort actions or on data from mixed high- and low-effort actions yielded much better classification accuracy than training the classifier on data from high-effort actions only. This finding suggests that the initial training of a classifier should use EMG data composed of varied muscle contraction levels or low effort level in order to enhance the stability of the EMG classifier with respect to variability in the level of effort of muscle contractions. The effort level change from high (training) to low (testing) greatly decreased the classification performance, compared to the effect of the effort change from low (training) to high (testing). This could be because high-effort muscle contraction not only shifts the mean of the distribution of the studied time-domain EMG features but also increases the features' variance within the classifier space.

Our results indicated that muscle fatigue fortunately had a minor effect on all features except for the EMG histogram feature. However, when sets of combined features were used, selection of an appropriate feature combination was critical to compensate for the influence of muscle fatigue on classification performance. This is because the use of the optimal feature set with respect to muscle fatigue provided a significantly more robust classification (higher accuracy and less variation) across subjects than the use of the least stable feature set.

One of our results contradicts the conclusion of previous work with respect to the utility of the EMG histogram feature for pattern recognition. This previous study [12] showed that the EMG histogram feature was an effective feature that maintained the most separability between classes among all studied EMG features and was recommended by the authors for myoelectric artificial arm control. In our analysis, a consistent result was demonstrated only when no disturbance was present in the testing data. Without any perturbations, the EMG histogram feature produced the highest classification accuracy and was one of the four best-performing features. However, when the three studied perturbations were introduced, the EMG histogram feature was the least stable feature. Including the three disturbances in the testing data drastically reduced the classification performance of the EMG histogram feature. Although the EMG histogram feature appeared in the most stable feature set with respect to electrode location stability, this combination did not elicit a significant difference in classification accuracy when compared with the performance of the least stable feature set. In addition, the EMG histogram feature was not included in the most stable set with respect to effort, muscle fatigue, and combined stability analysis. Therefore, our study indicates that the EMG histogram feature is not robust, which challenges its value for the clinical implementation of EMG pattern recognition.

One noticeable trend in our results is the robust performance of *AR *and *Ceps *features. When there was no disturbance in the testing data, both features extracted sufficient neural information. In the effort, fatigue, and combined stability analyses, the most stable feature sets shared *AR *and/or *Ceps *features. Nevertheless, *AR *and *Ceps *features are multi-dimensional; inclusion of these features in a feature set increases the dimensionality of the feature vectors, which increases the complexity of the classifier and the computational burden for real-time prosthesis control. Hence, there is a trade-off between feature stability and the computational efficiency of classification. The three feature sets that we identified as optimal overall (Figure 5) included *waveLen*, *AR*, and *Ceps*, resulting in a 20-dimension feature per EMG channel. When the number of EMG channels increases, dimensionality reduction of the feature vector following feature extraction might be necessary to improve computational efficiency. Additionally, the dimensionality we included for the multi-dimensional features (nine) was based on reports in the literature [12]. Determining the optimal dimensionality of features such as *Ceps *and *AR *is the logical next step in this investigation.

There are several limitations in this study. First, we limited our study to time-domain features. The implementation of frequency and time-frequency domain features with other types of classifiers is worth investigating in the future. Another limitation is that muscle fatigue during use of EMG-based HMIs is mainly due to repetitive, long-term muscle use; the muscle fatigue in this study was intensity-induced (short-term) because of the time constraints for conducting the experiments. Additionally, because the level of muscle fatigue was not controlled across subjects, some subjects demonstrated very low level of muscle fatigue and slight changes in EMG signals.

Based on the results of this study, relying only on "optimal" EMG features is not sufficient to overcome the variations in EMG signals caused by the considered disturbances. Additional solutions, such as effective training strategies and adaptive pattern recognition [16], are promising, although the challenges in making these potential solutions clinically viable still exist. Continued engineering effort is demanded towards development of a practical and robust HMI based on EMG pattern recognition.

## Conclusion

In this study we examined the influence of EMG signal changes elicited by electrode shift, changing amounts of user effort during muscle contraction, and muscle fatigue, on various time-domain EMG features and their resulting classification accuracies. Our results showed that the use of at least four combined EMG features enhanced the classifier performance, and multi-dimensional features, such as autoregression coefficients and cepstrum coefficients, were of greatest value. Although this study suggests three EMG feature sets that could offset the impact of the three studied disturbances, simple selection of these feature sets for EMG pattern recognition cannot fully solve the problem. Continuous efforts, such as developing effective classifier training strategies or physically fixing the electrode contact locations, are required to circumvent these undesired disturbances and minimize their effects on EMG pattern classification.

## Appendix: Linear Discriminant Analysis (LDA)

The idea of discriminant analysis is to classify the observed features to the movement class in which the posteriori probability *P*(*C*_*g *_|) can be maximized. *Cg *(*g*∈[1, G]) denotes the movement classes;  is the feature vector in one analysis window. The posteriori probability is the probability of class *Cg *given the observed feature vector  and can be expressed as(12)

where *P*(*C*_*g*_) is the priori possibility, *P*(|*C*_*g*_) is the likelihood, and *P*() is the possibility of observed feature vector . Therefore, the discriminant analysis-based classifiers can be mathematically described as(13)

Given movement class *C*_*g*_, the observed feature vectors have a multivariate normal (MVN) distribution, i.e. *P*(|*C*_*g*_) ~ *MVN*(*μ*_*g*_, Σ_*g*_), where *μ*_*g *_is the mean vector and Σ_*g *_is the covariance matrix of the class *C*_*g*_. Additionally, assume that the priori possibility *P*(*C*_*g*_) is equivalent for each movement class, and every class shared a common covariance, i.e. Σ_*g *_= Σ. Hence, the maximization of posteriori possibility in (13) becomes(14)(15)

is defined as the linear discriminant function.

In the offline training *μ*_*g *_and Σ were estimated by feature vectors calculated from a large amount of training data and were stored in the flash memory.

where *Kg *is the number of observations in class *C*_*g*_;  is the *k *observed feature vector in class *Cg*; *Fg *is the feature matrix ; *Mg *is the mean matrix  that has the same number of columns as in *Fg*. Therefore, the parameters in the linear discriminant function (15) were known, i.e.(16)

In the testing phase, the observed feature  derived from each analysis window was fed to the classifier to calculate  in (16) for each movement class and was classified into a specific class  that satisfied

## Competing interests

The authors declare that they have no competing interests.

## Authors' contributions

DK designed experiments, conducted data collection and analysis, and drafted the manuscript. HH and TK supervised the study and revised the manuscript. All authors read and approved the final manuscript.
